# Determining the response of African biota to climate change: using the past to model the future

**DOI:** 10.1098/rstb.2012.0491

**Published:** 2013-09-05

**Authors:** K. J. Willis, K. D. Bennett, S. L. Burrough, M. Macias-Fauria, C. Tovar

**Affiliations:** 1 Long-term Ecology Laboratory, Biodiversity Institute, Department of Zoology, University of Oxford, Oxford OX1 3PS, UK; 2 Department of Biology, University of Bergen, PO Box 7803, 5020 Bergen, Norway; 3 School of Geography, Archaeology and Palaeoecology, Queen's University Belfast, Belfast BT7 1NN, UK; 4 Department of Earth Sciences, Uppsala University, 752 36 Uppsala, Sweden; 5 School of Geography and the Environment, University of Oxford, South Parks Road, Oxford OX1 3QS, UK

**Keywords:** Africa, ecosystem services, climate change, aridity, precipitation, palaeoecology

## Abstract

Prediction of biotic responses to future climate change in tropical Africa tends to be based on two modelling approaches: bioclimatic species envelope models and dynamic vegetation models. Another complementary but underused approach is to examine biotic responses to similar climatic changes in the past as evidenced in fossil and historical records. This paper reviews these records and highlights the information that they provide in terms of understanding the local- and regional-scale responses of African vegetation to future climate change. A key point that emerges is that a move to warmer and wetter conditions in the past resulted in a large increase in biomass and a range distribution of woody plants up to 400–500 km north of its present location, the so-called greening of the Sahara. By contrast, a transition to warmer and drier conditions resulted in a reduction in woody vegetation in many regions and an increase in grass/savanna-dominated landscapes. The rapid rate of climate warming coming into the current interglacial resulted in a dramatic increase in community turnover, but there is little evidence for widespread extinctions. However, huge variation in biotic response in both space and time is apparent with, in some cases, totally different responses to the same climatic driver. This highlights the importance of local features such as soils, topography and also internal biotic factors in determining responses and resilience of the African biota to climate change, information that is difficult to obtain from modelling but is abundant in palaeoecological records.

## 1. Introduction

Tropical and subtropical African biomes, in particular forests and savannas, have long been recognized for their important ecosystem services and associated human benefits. African forests are valued for both their importance in terms of regulating (e.g. CO_2_ drawdown [1]) and provisioning services (e.g. timber, non-timber forest products [2]) and savannas for their potential in terms of provisioning and cultural services, for example, grazing land for both animal husbandry and ecotourism [3]. Most recently, these African landscapes have also been highlighted in terms of their provisioning services for biofuels [4]. Determination of these current ecosystem services at regional and landscape scales and the valuation of these benefits is the focus of much research presently (see examples in the electronic supplementary material, table S1). However, it is also recognized that these ecosystem services are changing in response to changes in climate and this will have a significant impact on provision of services in the future [5]. A key research challenge is to quantify the impact of these forecasted climatic changes on the African tropical and subtropical biota which are important for current and future ecosystem service provision.

There are currently two main approaches to assess the impact of future climate change on African biota: (i) modelling the response of species and/or vegetation functional units to changes in climate, i.e. using the present distribution of biota/climate to develop models to predict future change; and (ii) analysing the palaeo-record, i.e. to examine past biotic responses to climate changes analogous to forecasted climate to determine rates and possible direction of future responses to climate change.

Modelling approaches to assess the impact of future climate change on biota include bioclimatic envelope models and dynamic vegetation models. In the first, present-day distribution of the species in relation to the climatic envelope is calculated, and this climate/distributional range is then used to predict future range shifts in response to climate change (for a review, see [6]). In Africa, a number of studies have been carried out using this approach, indicating that for some species, significant loss of suitable climate space will result in extinctions. For example, a modelling study of 227 African mammals using climate predictions for 2050 and 2080 from the HadCM3 general circulation model (Intergovernmental Panel on Climate Change (IPCC) special report on emissions scenarios A2 and B2 [7]) indicated that between 25 and 40% of the species modelled will be critically endangered or extinct by 2080 [8]. Similarly, modelled range shifts for 5197 sub-Saharan plant species, using the same general circulation model, predicted the extinction of between 25 and 42% of species by 2085 due to reduction in suitable climate space, especially in the Guineo-Congolian forest [9]. It is widely acknowledged, however, that these models are highly sensitive to the algorithms used [10,11]. Moreover, they are unable to robustly quantify vegetation responses to conditions outside the range of variability of their training datasets (i.e. non-analogous [12–14]), such as future high CO_2_ atmospheric concentration or temperatures not observed currently. Differences in the assumptions made regarding non-analogous conditions might result in virtually opposite forecasts of extinction risks [15]. Other problems include an often poor understanding of the full ecological tolerances of the modelled plants/animals, determination of the full spectrum of biotic interactions, and coarse spatial scales (approx. 100 km grid squares) [6].

Dynamic vegetation models surmount many of the above-mentioned limitations, as they are mechanistic and explicitly formulate physiological, biophysical and biogeochemical processes as well as species interactions [16], and can thus be used to predict ecosystem response in non-analogous scenarios [17–19]. In particular, coupled vegetation dynamics and climate models have been used to demonstrate the importance of vegetation and land-cover feedbacks in the strength of the African monsoon over West Africa [20–22]. Despite their great insights and usefulness, the complexity of dynamic vegetation models means that they are very often used with plant functional types (PFTs) instead of species (e.g. tropical broadleaved evergreen, temperate needle-leaved evergreen, etc.) [19,23] and at coarse resolution: as a result, the use of PFTs does not enable these models to account for the observed individual species responses to environmental changes [24].

The use of historical and palaeo-records to determine possible biotic responses to future climate change has been less used to date. This is, in part, due to perceived circularities stemming from the use of proxies such as fossil pollen as both climatic and biological sources of information. The introduction of new tools and proxies in recent years has, however, resulted in a growing body of palaeoclimate-proxy evidence that is independent of the fossil data detailing biotic response (table 1). This paper presents a review of some of these newly emerging datasets alongside the fossil evidence for biotic responses to examine the insights that these palaeo-records can provide on the potential responses of African biota to projected future changes in climate. It focuses on three regional forecast climate scenarios in Africa, namely enhanced wetness, enhanced aridity and faster rates of climate warming. Fossil evidence for spatial variations in biota in response to climate change in Sahara, West Africa, East Africa and Southern Africa is examined and then discussed in the context of the information that they provide for determining current and future changes of biota to predicted climate changes.

**Table 1.** Temperature and wetness phases in Africa over the past 25 000 cal year BP, based on palaeo-proxies. Information of location and references for each record and the proxies used is detailed in figure 1 and the electronic supplementary material, table S2 following the number attached to each site name. Where absolute values are provided in the source publications, these are given in the electronic supplementary material, table S3. All other proxy interpretations are taken directly from the original source data. ‘isotopes’: isotopic and molecular climate proxy data; 'mixed': pollen (biotic response) included.

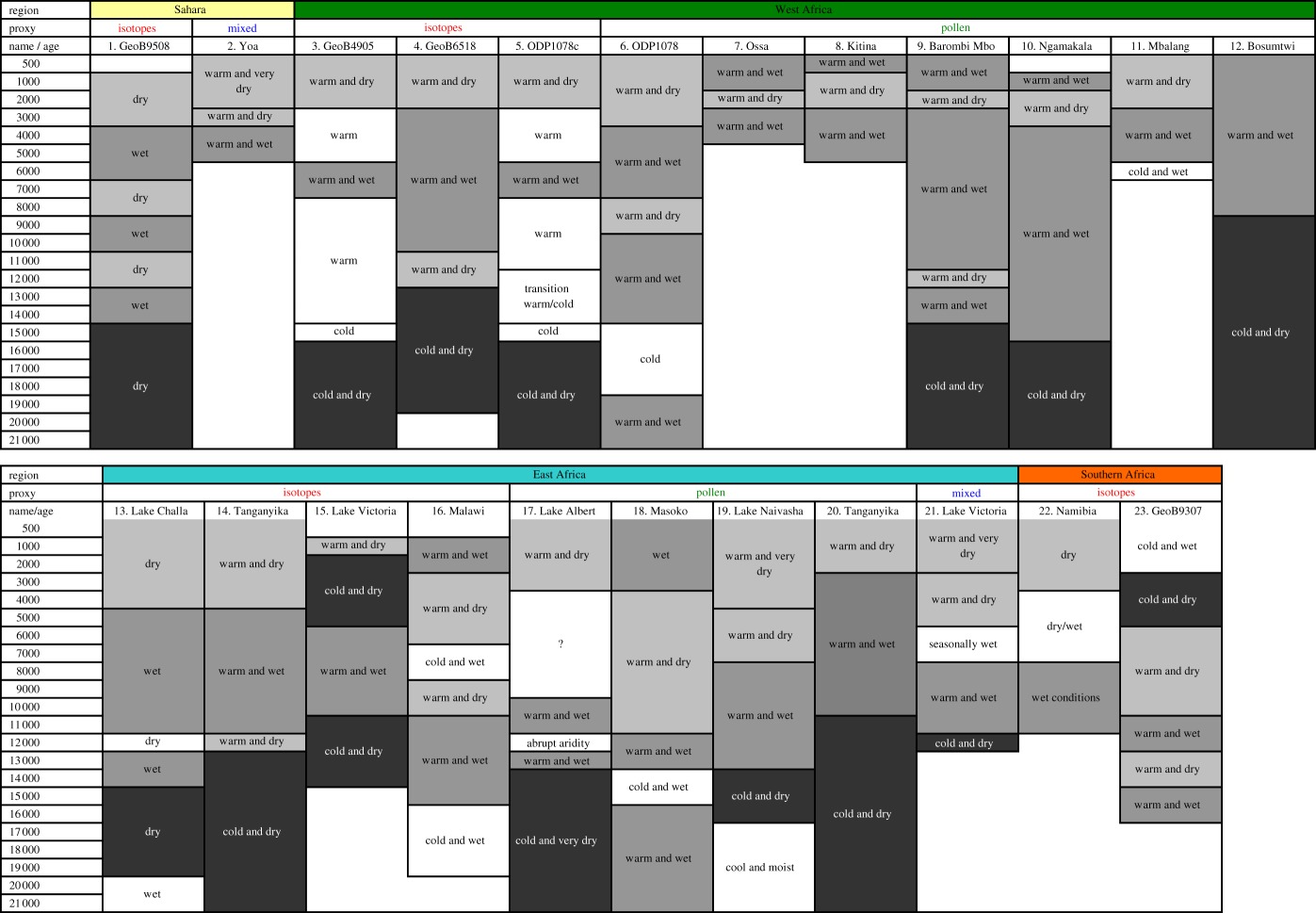

## 2. Current and future climate change in tropical and subtropical Africa

**Figure 1.**
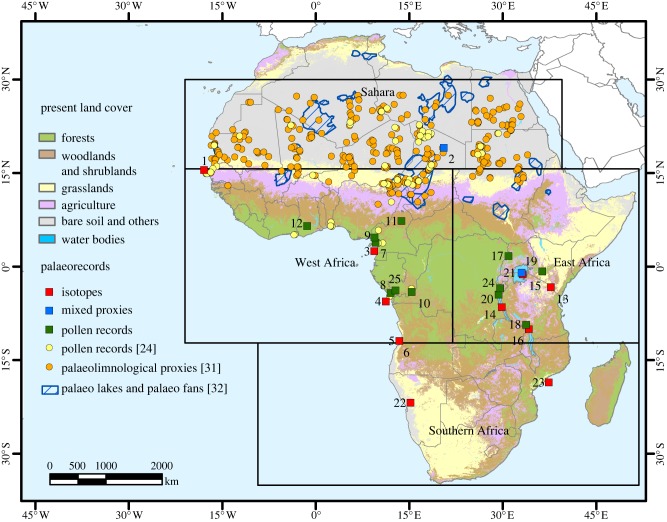
Location of the main palaeo-records in Africa. Squares: palaeo-records explicitly mentioned in the text (numbers as in table 1). Names and description in the electronic supplementary material, table S2. Mixed proxies involved a combination of pollen, molecular proxies and/or diatoms. Circles: records from other palaeo-record compilations mentioned in the text [24,31]. Main water bodies during the African humid period obtained from [32]. Present land cover based on [33]. African regions based on [25].

In order to simplify the great diversity of climates across Africa, we have used the regional division used by the IPCC [25] to narrow the focus of our review, namely to consider records from Sahara, West Africa, East Africa and Southern Africa (see the electronic supplementary material, figure S1). Near the equator, within the inter-tropical convergence zone (ITCZ), high precipitation (with equinoctial maxima) is mostly modulated by insolation, and regional variability is due to the influence of orography and oceans. North and south of this region, climate is dominated by the African monsoon system ([26]; see the electronic supplementary material, figure S1). Subtropical West Africa experiences one rainy season (May to September), modulated to a great extent by El Niño–Southern Oscillation (ENSO), sea surface temperatures (SSTs) over the Atlantic, and strong land–atmosphere interactions [27,28]. Subtropical East Africa in comparison experiences two rainy seasons, the ‘long rains’ (March to May) and the highly variable ‘short rains’ (September to December) [29,30], driven by the seasonal migration of the ITCZ and the movement of the Congo Air Boundary (figure 1) [34]. Finally, Southern African climate is highly variable regionally and modulated by the Atlantic and Indian oceans [35–38] as well as ENSO [39,40].

Given the diversity of the current climate in Africa, it is not surprising that climate predictions for this continent are also highly complex. From the most recent IPCC records, the following can be summarized for changes to the African climate by the end of this century (2080–2099):

— precipitation forecasts indicate high regional variability within Africa; some regions will get drier and other regions wetter. In East Africa, precipitation will probably increase during the ‘short rains’ season (October to December; [30,41]). In comparison, Southern Africa's climate is robustly predicted to get drier [42], especially in the austral winter [43] and to a lesser degree in spring and summer [44]. The Sahara is expected to stay dry or become drier [45]. Finally, in the Sahel and West Africa it could get wetter or drier [21,46–48], because despite recent improvements in the coupled model inter-comparison project phase 5 [49], there is still substantial inter-model spread in model projections (especially in the duration and intensity of the rainy season in the Sahel [27,50,51]);
— rate of climate change will increase. James *et al.* [52] report high inter-model agreement (from a total of 24 global climate models, GCMs) in predictions on faster warming rates over Africa than the global average; and
— temperature projections from CIMP5 [53] for the late-twenty-first century indicate warming in all months of the year in all African regions. Scenario-dependent (ranging from RCP2.8 to RCP8.5 [53]) temperature increases range from approximately 1.0°C to 4.4°C and in some cases 5.1°C in all African regions (as defined in [25]) and in both winter and summer months.

## 3. Biotic responses to past climate conditions analogous to projected scenarios

Fossil proxy climate evidence indicates that many of these predicted climatic changes for the African continent (warmer and wetter; warmer and drier; and increased rates of climate warming) of similar magnitudes have also been encountered in the past. Section 3*a* examines the evidence for these past climatic fluctuations and the biotic responses to them.

### (a) Warmer and wetter

There is now considerable independent palaeoclimatological evidence to suggest that warmer and wetter conditions occurred throughout many parts of western Africa, eastern Africa and the Sahara between approximately 11 000–4000 cal year BP, an interval commonly known as the African Humid Period (AHP) [17,31,54–60] (table 1). Current understanding of the causes of these conditions points towards a general response to changes in insolation, which in East Africa was associated with an increase in dry season precipitation (and thus a reduction in seasonality [17]) and in West Africa with an increased penetration of an enhanced west African monsoon [21], with increased overall precipitation.

Mapping and dating of palaeo-river channels, closed basins, palaeo-lake shorelines and spillways demonstrates that the vast Saharan region from approximately 11 000 to 8000 cal year BP contained a series of linked lakes, rivers and inland deltas comprising a large interlinked waterway, channelling water, plants and animals into and across it [31,32] (figure 1). In addition, deuterium/hydrogen isotopes of leaf waxes (proxy for wetness) indicate wetter conditions in Sahara and West Africa (table 1) [61,62], and alkenone-derived SST reconstructions (proxy for temperature) indicate regionally warmer conditions in West Africa [63] (table 1).

Palaeoecological evidence indicates that biotic responses to this interval of enhanced wetness were dramatic. In the Sahara and West Africa, pollen-based vegetation reconstruction from 73 sites (located between 4.3° S and 25.5° N; figure 1) [24] indicates a northward progression of many woody species into areas that are now classified as Saharan desert: a range expansion of up to 400–500 km northward of present range. This resulted in some tropical trees growing at 15°N in an area that is currently occupied by the Sahara desert [24]. Diversity reached a maximum at approximately 8.5 ka when Saharan/Sahelian elements which persisted throughout this period were mixed with savanna and forest species of Guineo-Congolian, Guineo-Sudanian and Sudanian affinities from the more humid south. All species studied indicate an individualistic rate and direction of movement, probably via water courses and gallery forests [24,32,64]. Thus, with this warmer and wetter climate, some regions that are now plantless hyperarid deserts were occupied by savanna, desert grassland [54,65] and in some places gallery forests [24], resulting in complex ‘non-analogue’ vegetation assemblages with mixed xeric–semimesic–mesic vegetation that has no modern counterpart.

In East Africa, palaeoenvironmental records including TEX86 (an organic palaeothermometer), deuterium/hydrogen and carbon isotope ratios of higher plant leaf waxes (proxy measures of palaeo-wetness) from Lake Tanganyika [60] (figure 1 and table 1) indicate that major changes in vegetation were highly correlated to climate trends during the wetter and warmer period 11 500–5000 cal year BP. Overall, the trend towards warmer and wetter conditions was paralleled by a shift in vegetation from one dominated by savanna and C_4_ grasses [66,67] to a landscape dominated by C_3_ trees and shrubs [66].

Significant, region-wide changes in vegetation therefore occurred in response to enhanced temperature and precipitation. These included large-scale range shifts, development of novel plant assemblages, higher concentrations of plant biomass in regions previously unable to support woody tree growth and extensive community turnover and assemblage change. However, the responses of plants appear to have been individualistic; there is little support, for example, for a vast ‘wave’ of forest migration—rather different species underwent range shift at different rates (varying in their response by thousands of years) and in different directions, and the overall response was nonlinear [24,32,64] and appears to have been strongly influenced by local abiotic factors.

### (b) Warmer and drier

Palaeoclimatic evidence indicates that a transition into a significantly drier climate occurred in some parts of Africa during the period 4000–1000 cal year BP (table 1). For example, in West Africa, deuterium/hydrogen isotopes from leaf waxes (proxy for wetness) and alkenone-derived SST reconstructions (proxy for temperature) indicate a decrease in wetness from 3200 cal year BP onwards and increase in temperature [63] (table 1).

In equatorial West Africa, one of the best records depicting biotic response to enhanced aridity is found in the fossil pollen sequence from Lake Mbalang, central Cameroon at approximately 7°N (figure 1) [68]. Around this site, palaeoecological evidence indicates there was a large-scale reduction in tropical forest, and the modern savanna became established around 3000 cal year BP (figure 1 and table 1). Lakes such as Sinnda completely dried up between 4000 and 1200 cal year BP [69]. Here, the structure of the forest changed significantly to favour dry-adapted semi-deciduous taxa, and disappeared altogether (4000–1200 cal year BP [69]) to the current grasslands. This forest response however was not uniform, with evidence from palaeoecogical records obtained from lakes Ngakamala, Kitina and Barombi Mbo (figure 1) indicating only the fragmentation of the forest and the appearance of savanna mosaics during the same interval of aridity [70–72]. Around Lake Ossa [73], there was a change in forest composition with an increase in pioneer species (*Alchornea* and *Macaranga*), whereas at Bosumtwi, almost no response is seen in the palaeo-record of vegetation [74]. This apparently local response of vegetation across West Africa to the same climatic forcing testifies to the importance of localized edaphic and geomorphic contexts and the variability in the stability and resilience of local vegetation structure to regional-scale climatic perturbations.

In East Africa, abrupt changes towards arid conditions are also recorded in the Tanganyika basin during the mid-Holocene, where an independent precipitation proxy (deuterium/hydrogen isotopes from plant waxes) indicates moderate drying from 6200 to 5500 cal year BP and a subsequent abrupt shift to arid conditions at 4900–4500 cal year BP [60]. Despite these rapid threshold-like shifts in hydrology, the vegetation record exhibited a gradual evolution towards C_4_ dominated grasslands from 11 000 cal year BP and then throughout the Holocene. At this site, xeric grassland/shrubland therefore showed an apparent strong internal ecosystem control, being to some extent resilient to observed climatic swings [60]. From around 2700 cal year BP to present, however, a reduction of arboreal species and expansion of the current grasses occurred [75].

Direction and timing of past vegetation change in response to warmer and drier climate in Africa was therefore often nonlinear, highly variable spatially and appears to have been dependent on specific ecosystem resilience to aridity at the local and regional scale. Assemblage change occurred in some areas, but there is little evidence to indicate a uniform change from forest to savanna or grassland; rather local factors seemed to strongly influence both the type and also timing of the response with some vegetation assemblages/areas displaying far greater resilience to enhanced aridity than others.

### (c) Faster rates of warming

IPCC GCMs indicate that Africa will undergo a net warming of 3–4°C by 2100, with mean predicted rates of warming more than 0.3°C per decade. These rates are a conservative number, because they are higher regionally (subtropics), under larger emissions scenarios and vary seasonally (i.e. June to July) [52,53]. A useful interval in time to examine past biotic responses to similarly rapid warming rates is during the transition coming into the present interglacial (for a review, see [76]). Palaeoclimatic estimates indicate that the rates encountered at this interval are roughly comparable with estimates of warming over the twenty-first century [76,77]. For example, at the higher latitudes of the Northern Hemisphere, the rapid warming at approximately 11.7 to 11.5 ka produced increases of 5°C and more over a few decades [78], and data from Greenland ice cores suggest that a more than 10°C warming may have occurred in a period of 20–60 years [79]. Evidence from Lake Masoko, East Africa (see the electronic supplementary material, figure S2), indicates an equally dramatic climate change coming out of the Younger Dryas, interpreted as a rapid increase in precipitation and/or temperature [80].

In East Africa, the most predominant response to this interval of rapid warming was ecological turnover and range adjustment. In the record from Lake Masoko [81], for example, a switch from forest dominated by taxa intolerant of a dry season to one containing species tolerant of a dry season occurred in less than 100 years at around 11 800 cal year BP (see the electronic supplementary material, figure S2). A similarly rapid turnover to climate warming is also seen in the palaeoecological record from Lake Kashiru in Burundi at this interval in time. Here, there was a switch from a grassland dominated ecosystem to woodlands [82] (see the electronic supplementary material, figure S2). Interestingly, at both sites, only a few local extinctions are noted associated with this rapid climatic transition. Instead, the main response was the rapid change in abundance of different taxa, a pattern similar to that seen throughout the Northern Hemisphere at this interval in time [76]. African plant species, at least in the regions studied thus far, appear to have had a level of tolerance and environmental plasticity to this interval of rapid climate change that enabled them to persist.

## 4. Discussion

The palaeoecological records presented here indicate some important additional factors that need to be taken into account when attempting to determine biotic response to future climate change; these are nonlinear features that will not necessarily be determined through modelling approaches alone, and can be summarized as follows.

First, there is a nonlinearity in the timing of the response of vegetation change to the same climatic driver. It would appear that slow climate trends can result in abrupt shifts in vegetation, and rapid climate changes may not incur rapid vegetation changes. For example, Tierney *et al.* [60] used paired compound-specific isotope records from the Holocene climate transition to show that major shifts in vegetation cover in the Tanganyika basin at 6.2–5.5 ka occurred during a time of only moderate drying. Prior to this, vegetation response was characteristically gradual, despite changes in wetness being typically abrupt [60]. This mismatch in the nature and timing of the relationship between climate variables and vegetation response is also noted in other regions of Africa [83]. Understanding this nonlinear behaviour is critical to understanding the level of confidence that can be placed in current and future predictions on the rate of response of African biota to climate change.

Second, there is nonlinearity in terms of the spatial response. In equatorial West Africa, for example, the interval of enhanced aridity between 4000 and 1000 cal year BP resulted in dramatic vegetation shifts from forest to savanna and to grassland in some areas, whereas other areas with the same climatic trigger saw little vegetation response. Reasons for these differences are important for determining spatial patterns of resilience across landscapes and, in particular, the sustainability of ecosystem service provision through time in response to climate change.

Third, there is nonlinearity in terms of the response of individual species and communities to climate change. In response to the AHP, for example, a large number of datasets from across West Africa indicate that while many woody species migrated north leading to the ‘greening of the Sahara’, they did so at individual rates and often following their own individual spatial trajectories, influenced by local topographical conditions [24,32,64]. Evidence for a large ‘wave-like’ biome movement in response to enhanced precipitation is totally lacking. These individual diffuse patterns of movement across landscapes in the fossil record, resulting in novel species assemblages and mosaic vegetation communities, provide a realistic representation of vegetation movement across the African landscape that could provide an important framework for future modelling studies.

All of the above is very important information for the current and future management of African landscapes because while at the broadest scale we might see extrinsic forcing leading to widespread turnover, the vast majority of evidence emerging from fossil records suggests that the rate and nature of this change is spatially highly variable and nonlinear. To date, palaeoecological data have provided qualitative information on the regional envelope of environmental variability for use in modelling [84], but it has enormous potential to be used in a quantitative sense to validate and hindcast models that project responses forwards into the future from the biome to the species scale [85]. The difficulty of obtaining the required lengthy, high temporal resolution data series that is required by such models may potentially be overcome by using combinations of high-resolution palaeoecological data and independent climate records during key periods analogous to forecast changes in climate.

Two other sources of information that are often not considered in modelling approaches but can be obtained from historical and palaeoecological records are the legacy of human impact and increasing atmospheric CO_2_ concentrations on the African landscapes. Both have clearly had an impact and continue to have a significant impact on African biota, and the potential for using a longer-term perspective to obtaining information necessary for modelling of these two drivers will briefly be discussed in the following section.

Predicted future levels of anthropogenically induced atmospheric CO_2_ indicate values ranging from 467 to 567 ppmv by 2050 and from 540 to 970 ppmv by the end of the twenty-first century. Ranges result from using different emission socio-economic scenarios [7,53]. Increasing CO_2_ combined with warming affects photosynthesis and plant productivity, in what is known as the CO_2_ fertilization process [76,86–88]. When the fertilization effect is implemented in dynamic vegetation models for future climatic scenarios, the output predicts enhanced plant growth, an increase in ecosystem productivity and higher diversity [89]. Historical records based on long-term monitoring plots, remote sensing and forest inventories largely agree with these predictions [90,91]. In the savanna biome of Southern and Eastern Africa, for example, historical photographs over the past 100 years indicate increased woody thickening of the savanna [13,92,93]. Evidence indicates that this thickening is due to the CO_2_ fertilization process, through a mechanism of which fire survival and suppression is a key component [12,13,93–95]. Moreover, the observed increase in woody shrubs and trees in these savannas has involved a reduction of C_4_ grasses in the understorey, implying fundamental changes of the species and functional assemblage of these communities [96]. This agrees with theorized increased competitiveness of trees and C_3_ plants in general under increased atmospheric CO_2_ concentrations [97]. To add further support to this suggestion, *Acacia karroo* and *Terminalia sericea*, typical species of African savanna, were grown in growth chambers using a gradient of CO_2_ treatments including that at the last glacial maximum (180 ppm) and above-ambient (450 ppm). In both species, as CO_2_ concentration was increased, there was a significant increase in the mass of the root and shoot material [95].

Palaeoecological and historical records can also provide important additional information to understand impacts of future human activities upon the landscape. For example, changes in savanna fire regime of Southern Africa have been observed as a result of agro-pastoralism, especially in the past 4000 years, which may have potentially affected forest expansion [98]. In addition, in central West Africa, a recent study [99] has challenged the common belief that an arid event at around 2500 cal year BP caused a major decrease in forest [100–102], proposing instead that human land-use change was responsible for this vegetation change. Although this hypothesis remains under debate [103,104], it highlights the fact that more attention should be paid to past human impacts and their legacy on the current vegetation/climate of tropical Africa. This is a point often missed in modelling approaches and something that future studies should address when considering the relationship between vegetation and climate in Africa.
